# GW-Bodies and P-Bodies Constitute Two Separate Pools of Sequestered Non-Translating RNAs

**DOI:** 10.1371/journal.pone.0150291

**Published:** 2016-03-01

**Authors:** Prajal H. Patel, Scott A. Barbee, J. Todd Blankenship

**Affiliations:** 1 Department of Biological Sciences and Eleanor Roosevelt Institute, University of Denver, Denver, Colorado, United States of America; 2 Molecular and Cellular Biophysics Program, University of Denver, Denver, Colorado, United States of America; University of Valencia, SPAIN

## Abstract

Non-translating RNAs that have undergone active translational repression are culled from the cytoplasm into P-bodies for decapping-dependent decay or for sequestration. Organisms that use microRNA-mediated RNA silencing have an additional pathway to remove RNAs from active translation. Consequently, proteins that govern microRNA-mediated silencing, such as GW182/Gw and AGO1, are often associated with the P-bodies of higher eukaryotic organisms. Due to the presence of Gw, these structures have been referred to as GW-bodies. However, several reports have indicated that GW-bodies have different dynamics to P-bodies. Here, we use live imaging to examine GW-body and P-body dynamics in the early *Drosophila melanogaster* embryo. While P-bodies are present throughout early embryonic development, cytoplasmic GW-bodies only form in significant numbers at the midblastula transition. Unlike P-bodies, which are predominantly cytoplasmic, GW-bodies are present in both nuclei and the cytoplasm. RNA decapping factors such as DCP1, Me31B, and Hpat are not associated with GW-bodies, indicating that P-bodies and GW-bodies are distinct structures. Furthermore, known Gw interactors such as AGO1 and the CCR4-NOT deadenylation complex, which have been shown to be important for Gw function, are also not present in GW-bodies. Use of translational inhibitors puromycin and cycloheximide, which respectively increase or decrease cellular pools of non-translating RNAs, alter GW-body size, underscoring that GW-bodies are composed of non-translating RNAs. Taken together, these data indicate that active translational silencing most likely does not occur in GW-bodies. Instead GW-bodies most likely function as repositories for translationally silenced RNAs. Finally, inhibition of zygotic gene transcription is unable to block the formation of either P-bodies or GW-bodies in the early embryo, suggesting that these structures are composed of maternal RNAs.

## Introduction

Processing body (P-body) associated RNAs constitute a subset of non-translating RNAs present in a cell [1]. These RNAs cycle actively between translating ribosomes and P-bodies, where they either undergo sequestration or decay [2,3,4,5]. Compositionally, P-bodies are associated with the 5’-3’ exonuclease XRN1/Pcm as well as decapping factors DCP1 and DCP2, and decapping modulators Pat1/Hpat, Dhh1/Me31B, and the LSm1-7 complex [2,6,7]. Based on this makeup, P-bodies have long been thought to be specialized sites of RNA degradation [6]. The movement of RNAs into P-bodies requires active translational repression [8,9]. The fact that translational repression of RNAs occurs prior to their localization into P-bodies indicates that RNA sequestration in P-bodies is not a primary mechanism for translational repression [8].

GW-bodies are similar to P-bodies as both have been shown to contain factors that promote RNA decapping and decay, including the decapping factor DCP1, decapping activator LSm-4, as well as XRN1/Pcm [10,11,12]. Due to this compositional similarity, GW-bodies have been hypothesized to be the higher eukaryotic counterpart of these structures [10]. In addition to these decay factors, GW-bodies also contain Gw/GW182, a protein which is not conserved in yeast [13]. Gw/GW182 encodes a large scaffolding protein containing an N-terminal domain composed of GW/WG motifs, an ubiquitin-associated domain (UBA), and an RNA recognition motif (RRM) [13]. The N-terminal GW/WG motif-bearing domain has been shown to bind to AGO1 while the C-terminus interacts with the CCR4-NOT deadenylation complex, implicating Gw/GW182 in coordinating microRNA-mediated silencing with RNA turnover [11,14,15,16]. In agreement with these biochemical observations, the microRNA-induced silencing complex (miRISC) component AGO1 as well as miRISC-targeted RNAs both localize to GW-bodies in tissue culture and *C*. *elegans* [9,11,17,18,19,20]. Furthermore, the shuttling of miRISC-targeted RNAs to GW-bodies is important to effect gene silencing as AGO1 proteins that cannot localize to these structures fail to silence RNAs [21]. Functionally, Gw/GW182 has been shown to be an effector of microRNA-mediated gene silencing and is required downstream of AGO1, further bolstering the argument that GW-bodies play an important role in microRNA-mediated gene silencing [19,21,22] Thus GW-bodies of higher eukaryotes are different from P-bodies in that they also serve as sites for microRNA-dependent RNA silencing and turnover. This suggests that an additional level of coordinated RNA turnover occurs in higher eukaryotic GW-bodies.

Several reports indicate that GW-body dynamics differ significantly from P-body dynamics. Unlike P-bodies, GW-bodies present in HEp-2 cells have been shown to assemble and disassemble in response to the cell cycle, increasing in size during S and G2 phases and disintegrating during mitosis [23]. P-bodies have been shown to disassociate upon cycloheximide treatment due to the sequestration of RNAs in polysomes [2,3,24,25]. GW-bodies in certain experimental contexts have been reported to be resistant to cycloheximide treatment, indicating that they function in RNA storage and sequestration rather than decay [26]. Here we study the dynamics and composition of GW-bodies and P-bodies *in vivo* during early *Drosophila melanogaster* embryonic development. P-bodies have been shown to be important for the both translational regulation and RNA degradation during early *Drosophila* development [27,28]. Analysis of *Drosophila* gawky *(gw*) mutants indicates that this gene product is required during early embryonic development at the midblastula transition (MBT), when maternal control of development is transferred to zygotic control and maternally-deposited RNAs undergo destabilization [12]. Using live imaging to examine the dynamics of GW-bodies and P-bodies, we find that P-bodies are present throughout early embryonic development and that there is a burst of P-body formation following the MBT. GW-bodies, however, are not present during the early syncytial cycles but form *de novo* at the MBT. As observed by live imaging, the *Drosophila* CCR4 homolog Twin is not present in punctate structures, indicating that Twin is excluded from both P-bodies and GW-bodies. We find that Gw proteins do not incorporate into P-body structures, revealing that P-bodies and GW-bodies are distinct structures and most likely constitute separate pools of non-translating RNAs. We also find that AGO1 does not localize to GW-bodies, but instead localizes with the maternal RNA translation repressor Smaug. Using translation inhibitors puromycin and cycloheximide, we demonstrate the GW-bodies also exist in equilibrium with actively translating pools of RNAs. Finally, blockage of zygotic gene activation using the RNA polymerase II inhibitor α-amanitin shows that these newly derived P-bodies and GW-bodies originate from maternal RNAs.

## Materials and Methods

### *Drosophila* stocks

Flies were raised at 25°C on standard media. Fly stocks: *Oregon-R* was used throughout as the wild type strain. *Maternal* α*-tubulin-GAL4VP16* (D. St. Johnston) was used to drive pUASp-EGFP:Gw transgene. Fly lines *P{PTT-GB}me31B*^*CB05282*^, *P{PTT-GA}twin*^*CA06641*^, *P{PTT-GA}AGO1*^*CA06914*^, and *P{FLAG*.*HA*.*AGO2}* were obtained from the Bloomington Stock Center.

### Transgenes

To generate pUASp-EGFP:Gw, the full length Gw coding sequence was PCR amplified from the pAc5.1B-EGFP-DmGW182 plasmid (Addgene; [11]) using forward and reverse primers flanked with NotI and XbaI restriction sites respectively. PCR product was digested with NotI and XbaI and ligated into pUASp [29] to generate pUASp-Gw intermediate. PCR amplified EGFP sequence from pAc5.1B-EGFP-DmGW182 with flanking NotI restriction sites was digested and ligated into NotI digested pUASp-Gw to generate pUASp-Gw:EGFP. This construct was introduced into flies using P-element transformation.

### Spinning disc confocal microscopy and pharmacological treatments of embryos

Live imaging of embryos was conducted using a Yokogawa CSU10 spinning disc confocal microscope (Zeiss Observer.A1). Embryos were dechorionated in 4% bleach, washed with water, and mounted in halocarbon 27 Oil (Sigma). All live images were acquired in one minute intervals using Planapochrom 63X 1.4NA Oil objective. For pharmacological manipulations, embryos were lined up and glued to a coverslip, and desiccated for 12 to 13 minutes in a closed chamber containing drierite. Following desiccation, embryos were covered in halocarbon 700 oil (Sigma). Embryos were subsequently injected using a micromanipulator (Narishige, MN-151) with either water, 10,000 μg/mL puromycin (Santa Cruz Biotechnology), 1,000 μg/mL microbially sourced cycloheximide (Sigma), or 100 μg/mL α-amanitin (Santa Cruz Biotechnology) and imaged.

### Immunofluorescence staining, confocal microscopy, and image analysis

For whole mount immunostaining, embryos were dechorionated in 4% bleach solution, washed copiously with water, and transferred to heptane. An equal volume of 4% paraformaldehyde fixative in 1X PBS pH 7.4 was added. Fixation was allowed to proceed for either 20 or 55 minutes. Following fixation, embryos were either devitellinized with methanol or manually. Standard immunostaining procedures were used to label fixed embryos. Primary antibodies were incubated overnight at 4°C. Secondary antibodies were incubated for two hours at room temperature. The following antibodies were used: monoclonal mouse α-DCP1 1:1000 [30]; polyclonal rabbit α-GFP 1:1000 (Torrey Pines Biolabs, TP401); polyclonal guinea pig α-Gw 1:3000 [12]; polyclonal rabbit α-HA 1:1000 (Santa Cruz Biotechnology, sc-805); polyclonal rat α-Hpat 1:500 [31]; monoclonal mouse α-Not1 1:100 [32]; polyclonal guinea pig α-Smaug [33]; polyclonal rabbit α-Twin/CCR4 1:300 [34]; monoclonal rat α-Vasa 1:500 (Developmental Studies Hybridoma Bank). We used Alexa 546-phalloidin 1:200 (Molecular Probes) to image F-actin. Appropriate Alexa 488 or Alexa 568 secondary antibodies (Molecular Probes) were used at 1:500. Samples were mounted in ProLong Gold Antifade Mountant (Molecular Probes). All images of fixed samples were acquired using Olympus Fluoview FV1000 confocal laser scanning microscope (IX81) using a PlanApoN 60X 1.42NA Oil objective. Images were processed using ImageJ software and Adobe Photoshop. For colocalization analysis, a total of four to five apical sections, each from a different embryo, were analyzed. Apical sections were selected based on the size of the foci being analyzed. Using ImageJ, these images were thresholded and subjected to particle analysis to quantify particle number. The incidence of particle colocalization was determined visually with the aid of a colocalization finder ImageJ plugin. Pearson correlation coefficients (PCC) were measured using Just Another Co-localization Plugin (JACoP) [35].

## Results

### Gw-bodies form *de novo* in the early *Drosophila* embryo during cellularization

**Fig 1:**
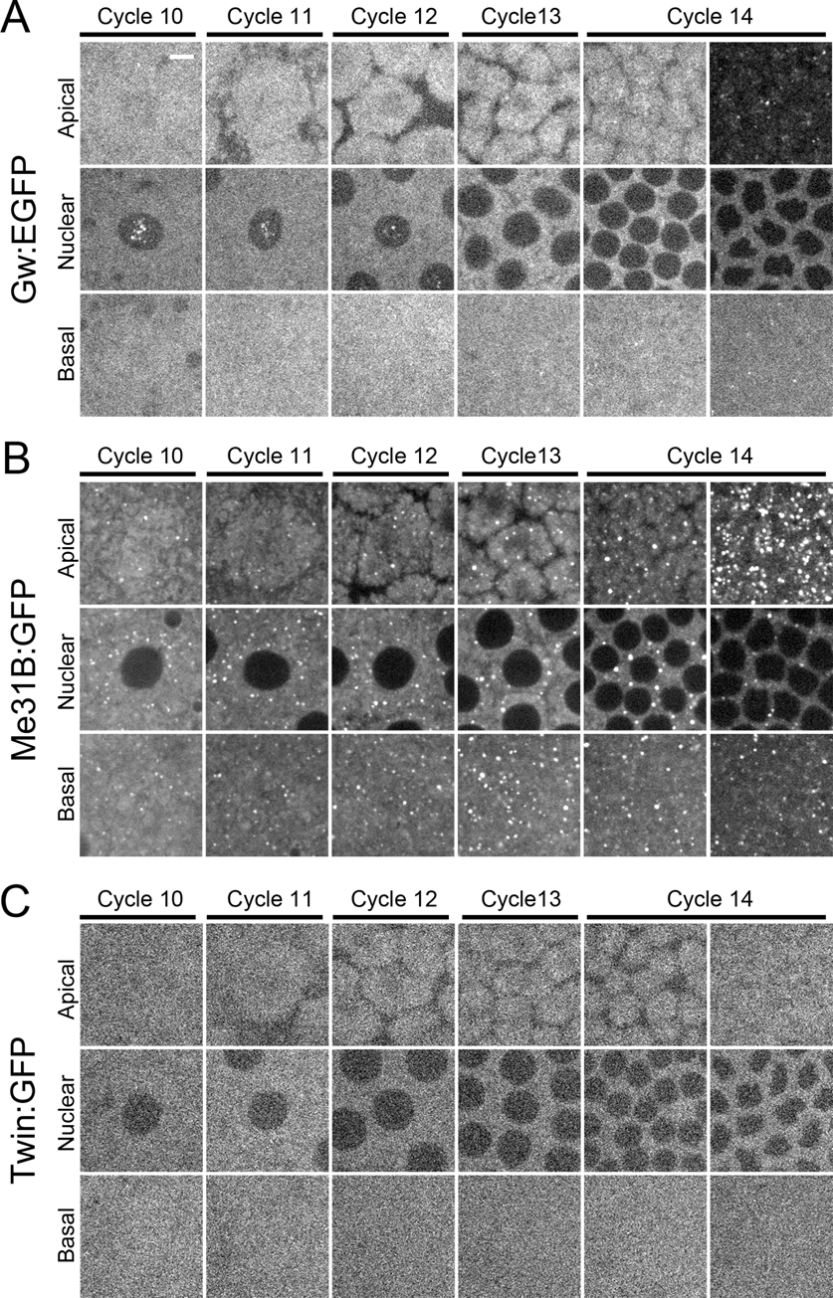
Cytoplasmic GW-bodies are absent during early embryonic development and form *de novo* at cellularization. Live imaging of Gw:EGFP, Me31B:GFP, and Twin:GFP during cortical syncytial cycles and cellularization. Apical, nuclear, and basal images of embryos during cycles 10, 11, 12, 13, and 14. Cycle 14 images were taken just after completion of cycle 13 (left image) or during cellularization (right image). (A) Embryos expressing Gw:EGFP. Gw is present in the nucleus during cortical syncytial cycles. Cytoplasmic GW-bodies form primarily during cellularization. (B) Embryos expressing Me31B:GFP. Me31B labeled P-bodies are present throughout cortical syncytial cycles. New P-body structures form in large numbers during cellularization. (C) Embryos expressing Twin:GFP. Twin is not associated as punctate structures. Scale bar = 5 μm.

To visualize GW-body dynamics in the early embryo, we generated and expressed a *UAS-Gw*:*EGFP* transgene under the regulation of the *maternal α-tubulin-GAL4VP16* driver. Gw:EGFP localization in these embryos is coincident with labeling by an antibody that recognizes the Gw protein (S1A–S1F Fig). We find that Gw:EGFP possesses two different subcellular localizations during syncytial cycle 10, localizing both to the cytoplasm as well as to foci within nuclei (Fig 1A, S1G–S1I Fig). The observation of nuclear Gw foci was highly surprising and has not been reported previously. Cytoplasmically localized Gw proteins at cycle 10, however, are not associated in foci. As syncytial cycles progress, a modest number of cytoplasmic Gw foci begin to form up to the start of cycle 14, accompanied by a concomitant decrease of nuclear Gw foci (Fig 1A). At late cycle 14 during cellularization, prominent cytoplasmic Gw foci become present in apical sections, and nuclear foci are virtually undetectable by live imaging (Fig 1A). Interestingly, this loss of nuclear foci correlates with the increase in cytoplasmic GW-body nucleation. Altogether, these results demonstrate that GW-body formation occurs *de novo* during syncytial cycle 14 when maternal RNAs undergo MBT-associated clearance from the embryo.

Proteins that regulate RNA decapping such as DCP1, DCP2, Hpat, and Me31B are standard markers for P-bodies [2,36,37,38,39,40]. As Me31B has been used previously to observe P-bodies in the early embryo [28], we used an endogenous Me31B protein trap line to follow P-body dynamics during syncytial divisions. Unlike Gw, Me31B proteins exist in prominent cytoplasmic puncta at cycle 10 (Fig 1B). Another major difference between Gw and Me31B puncta is that these Me31B puncta are not associated with nuclei (Fig 1B). Interestingly, Me31B puncta grow modestly in size and number as syncytial cycles progress but dramatically increase in number at cycle 14 during cellularization (Fig 1B). Thus, new P-body and GW-body formation both occur at a similar time point, suggesting that a common mechanism may regulate the formation of these structures. However, the low number of GW-bodies present prior to cellularization potentially indicates that GW-bodies and P-bodies in the early embryo are distinct structures.

Finally, we live imaged a Twin protein trap line. Twin/CCR4 encodes the catalytic subunit of the CCR4-NOT deadenylation complex [41,42]. In mammalian cells, Twin/CCR4 has been shown to localize to P-body structures [3,24]. We find that Twin localization is not focal but present uniformly throughout the cytoplasm (Fig 1C). Similar uniform, non-focal distributions of Twin have also been observed in Schneider 2 (S2) cells, suggesting that Twin/CCR4 is not a component of either P-bodies or GW-bodies either *in vivo* or *in vitro* (SAB, unpublished observation).

**Fig 2:**
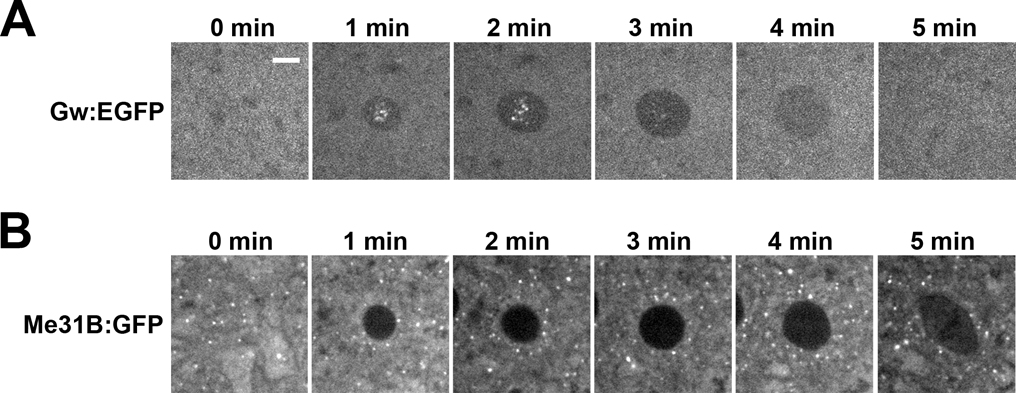
Nuclear GW-bodies assemble and disassemble with the cell cycle. Live imaging of Gw:EGFP expressing embryo during syncytial cycle 10. (A) Nuclear GW-bodies over a five minute time course of cycle 10. Nuclear GW-bodies peak in size and intensity and disassemble as the nucleus enters mitosis. (B) P-bodies are cytoplasmic and present throughout the cell cycle. Scale bar = 5 μm.

Previous studies of GW-bodies have suggested that the integrity of these structures alter with the cell cycle [23]. We observed a similar dynamic for nuclear GW-bodies during syncytial cycles. Syncytial nuclei undergo a modified, rapid cell cycle that alternates between mitosis and S phase with no G1 or G2 phase. We find that nuclear GW-bodies form and disintegrate as nuclei exit and enter mitosis (Fig 2A). P-body structures, on the other hand, are stable throughout the syncytial cell cycle (Fig 2B). Thus nuclear GW-bodies have a dynamic that is distinct from P-bodies.

### GW-bodies are compositionally distinct from P-bodies

**Fig 3:**
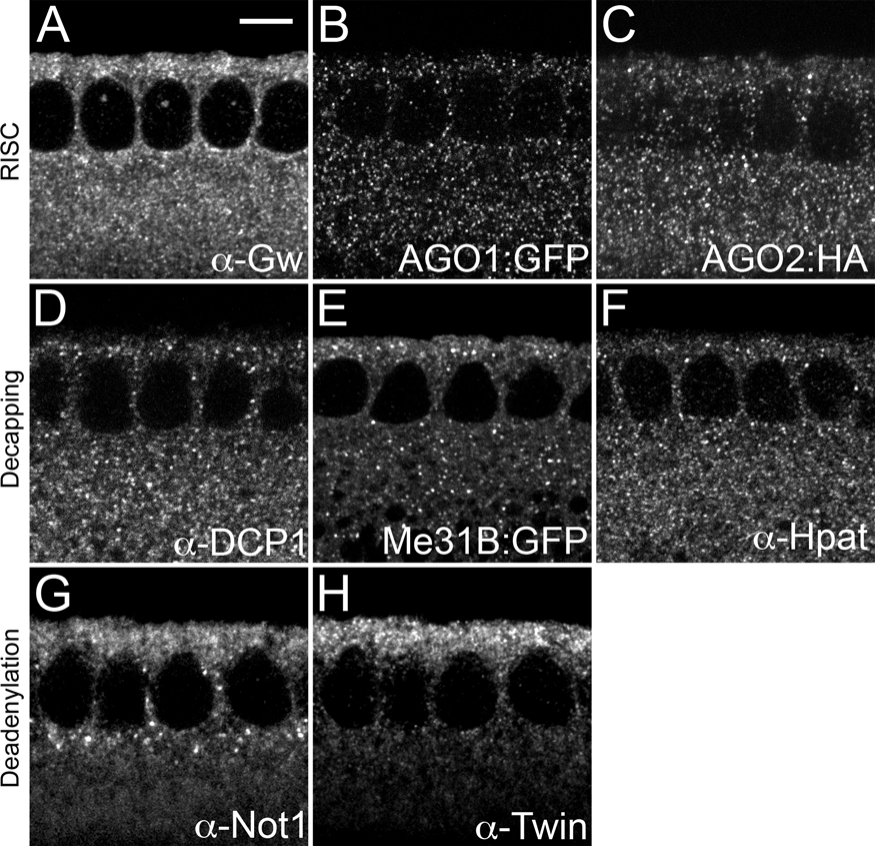
Distributions of RISC components, decapping factors, and deadenylation complex components during cycle 14. Sagittal views of embryos at cycle 14 labeled for (A) Gw, (B) AGO1, (C) AGO2, (D) DCP1, (E) Me31B, (F) Hpat, (G) Not1, and (H) Twin. RISC and decapping puncta are distributed throughout the cytoplasm, while deadenylation components are found enriched apically and around the nucleus. Nuclear Gw puncta are apparent in (A). Scale bar = 5 μm.

As Gw and Me31B positive foci are both present at cellularization, we investigated the distribution of GW- and P-body associated proteins at this stage. Gw has been shown to function in miRNA-mediated translational silencing in both mammalian and *Drosophila* cells [19,21,22]. We find that components of the RNA-induced silencing complex (RISC) such as Gw, AGO1, and AGO2 are present as cytoplasmic foci (Fig 3A–3C). These foci are not asymmetrically enriched in either an apical-basal or planar fashion. While nuclear GW-body foci are not detectable in live imaging experiments during cellularization, we were able to observe such foci using an anti-Gw antibody (Fig 3A). This immunohistochemical observation also indicates that nuclear Gw foci visualized by live imaging are not a consequence of protein overexpression. We find that P-body associated proteins such as DCP1, Me31B, and Hpat also exist as puncta that are distributed throughout the cell (Fig 3D–3F). However, proteins that function in regulating deadenylation are not uniformly localized. While foci containing RISC and decapping proteins are distributed throughout the cytoplasm, Not1 and Twin are both enriched around nuclei. Not1 is largely present apically, but also forms large foci basal to the nuclei (Fig 3G). Twin, however, is primarily enriched in the apical cytoplasm (Fig 3H). These two distinct localizations are highly surprising considering that Not1 and Twin function in a common deadenlyation complex and suggest that Not1 and Twin potentially exist in separate cellular structures. To test this directly we double labeled embryos for Twin and Not1. In apical sections where both proteins are enriched, Twin and Not1 foci do not co-localize (S2A–S2C Fig). We also find that large, basally located Not1 foci are adjacent to actin bundles (S2D–S2F Fig). Currently the function of these basal actin bundles is unknown. As Not1 and Twin function in the same protein complex, the lack of colocalization between Twin and Not1 suggests that the associations between these proteins are either transient or that Twin and Not1 function in several distinct complexes or that their interactions occur outside of foci. Altogether, we find that P-body components are present as foci during late syncytial cycles and cellularization.

**Fig 4:**
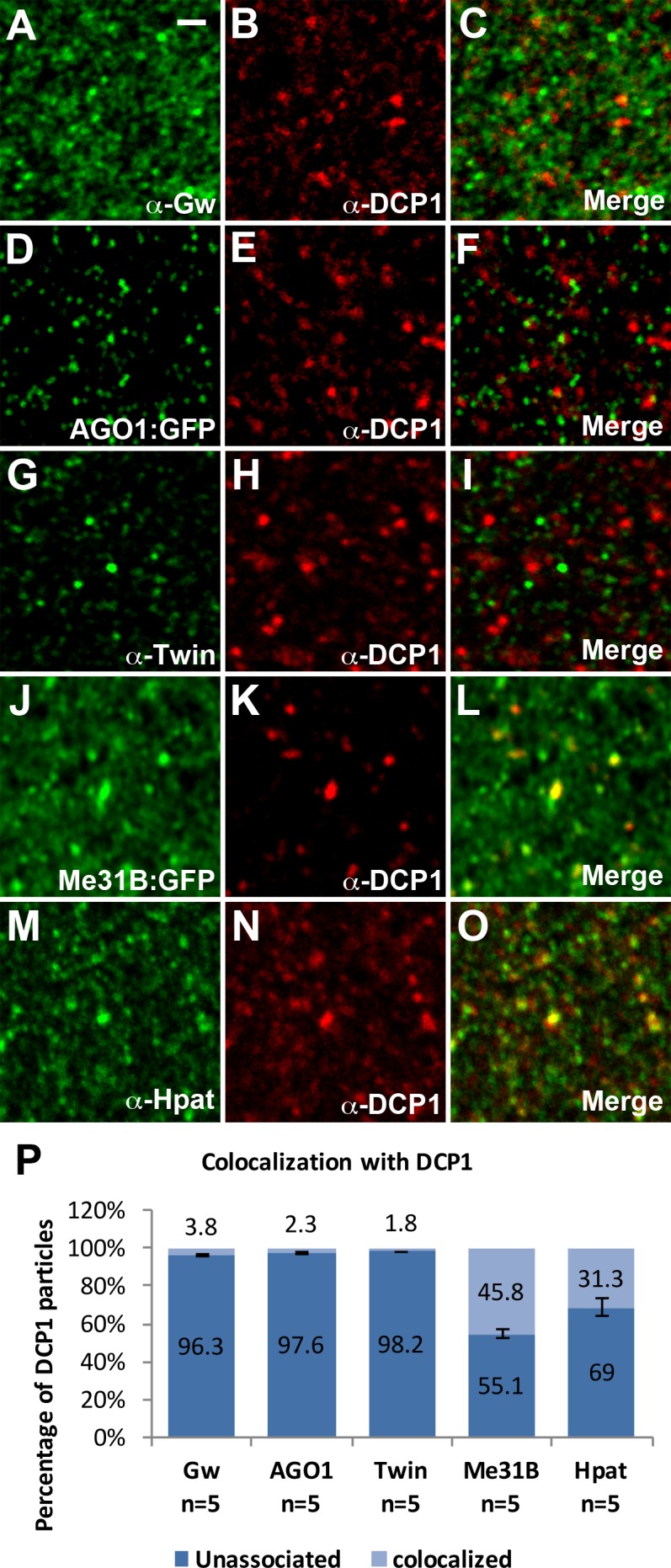
GW-bodies and P-bodies exist as separate structures. (A-I) DCP1 foci do not colocalize with the GW-body marker Gw (A-C), RISC complex component AGO1 (D-F), or the deadenylation enzyme Twin (G-I). (J-O) DCP1 colocalizes with decapping activators Me31B and Hpat. (P) Quantifications of colocalization with DCP1 foci. A total of 2429, 1909, 2248, 1583, and 1615 DCP1 particles were counted respectively for Gw, AGO1, Twin, Me31B, and Hpat double labelings with DCP1. The Pearson's correlation coefficient (PCC) value (mean ± SD) for Gw, AGO1, Twin, Me31B, and Hpat double labelings with DCP1 are .045 ± .020, .006 ± .003, .002 ± .008, .404± .076, and .238 ± .078 respectively. All images are from apical sections of cellularizing (stage 5) embryos. Scale bar = 1 μm.

Since GW-bodies in higher eukaryotes have been proposed to be analogous to P-bodies, one possibility is that Gw proteins associate with P-bodies during late syncytial cycles and cellularization. To determine if Gw foci observed during cellularization are P-bodies, we double labeled embryos with Gw and DCP1. We find that Gw and DCP1 did not colocalize, suggesting that GW-bodies and P-bodies exist as separate structures in the early embryo (Fig 4A–4C and 4P). We also find that DCP1 foci did not colocalize either with AGO1:GFP expressed from a protein trap line or with Twin (Fig 4D–4I and 4P). These data indicate that key components of miRISC are not present in P-bodies *in vivo*. The lack of association between Twin and DCP1 is consistent with our live imaging data. However, as expected, DCP1 does colocalize with decapping regulators such as Me31B and Hpat (Fig 4J–4O and 4P). Thus *Drosophila* P-bodies *in vivo* resemble P-bodies observed in yeast, which are typically composed of RNA decay factors.

**Fig 5:**
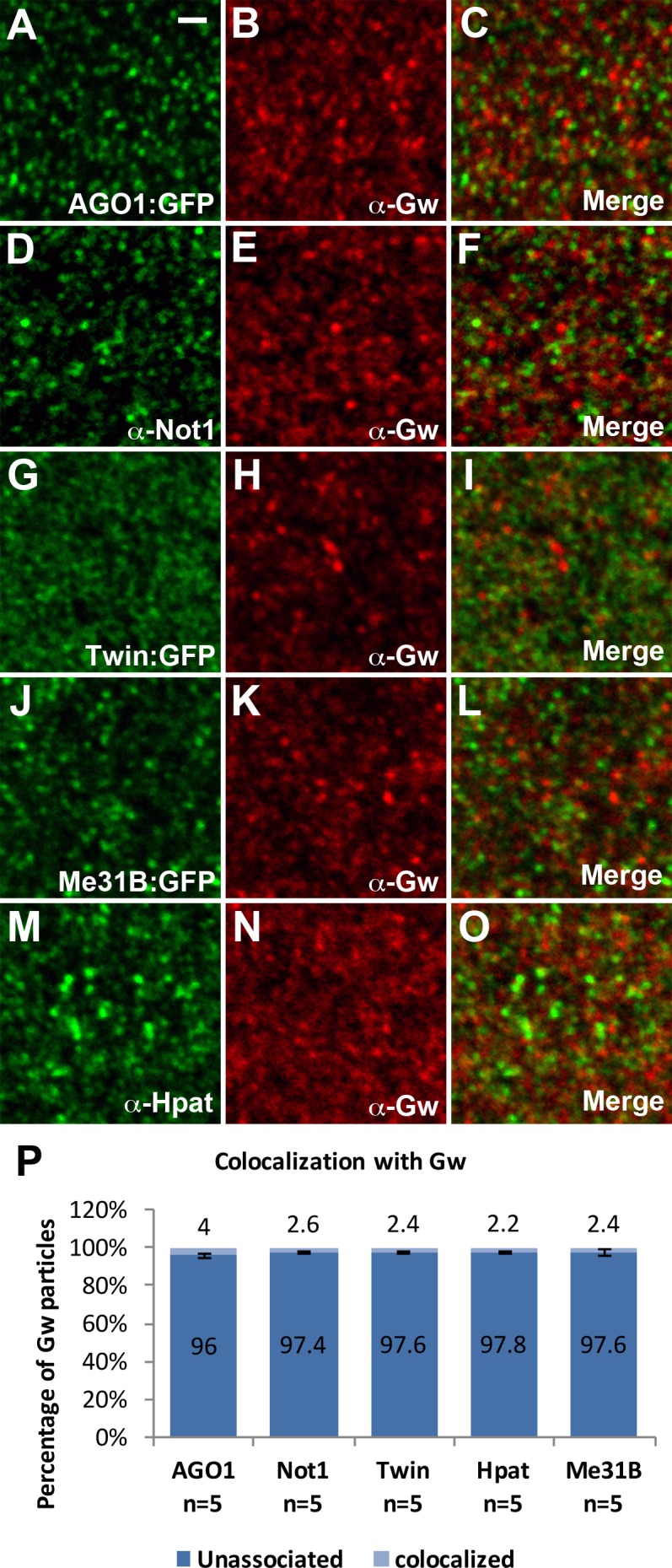
AGO1, deadenylation complex components, and decapping factors are absent from GW-bodies. (A-C) Gw foci do not colocalize with AGO1. (D-I) Gw foci also do not colocalize with deadenylation complex components Not1 and Twin. (J-O) Gw does not colocalize with decapping activators Me31B and Hpat. (P) Quantifications of colocalization with Gw foci. A total of 1608, 1801, 1858, 1440, and 1450 Gw particles were counted respectively for AGO1, Not1, Twin, Me31B, and Hpat double labelings with Gw. The Pearson's correlation coefficient (PCC) value (mean ± SD) for AGO1, Not1, Twin, Me31B, and Hpat double labelings with Gw are .011 ± .007, .017 ± .002, .010 ± .004, .013± .003, and .011 ± .006 respectively. All images are from apical sections of cellularizing (stage 5) embryos. Scale bar = 1 μm.

As Gw does not colocalize with the standard P-body marker DCP1, we wanted to determine if Gw associated with members of the miRISC complex. Unlike observations made in other experimental contexts [11,18], we find that AGO1 does not colocalize with Gw (Fig 5A–5C and 5P). The Gw protein has also been shown to physically interact with both Not1 and CCR4/Twin in mammalian and *Drosophila* cells [14,15,16]. We find, using double labelings of Gw with Not1 or Twin:GFP, that these proteins also do not localize to Gw foci in the early embryo (Fig 5D–5I and 5P). Furthermore, decapping proteins Me31B and HPat also do not colocalize with Gw (Fig 5J–5O and 5P). Altogether, these data suggest that GW-bodies in *Drosophila* embryos do not share a similar composition to P-bodies.

### Analysis of developmental RNA binding proteins

**Fig 6:**
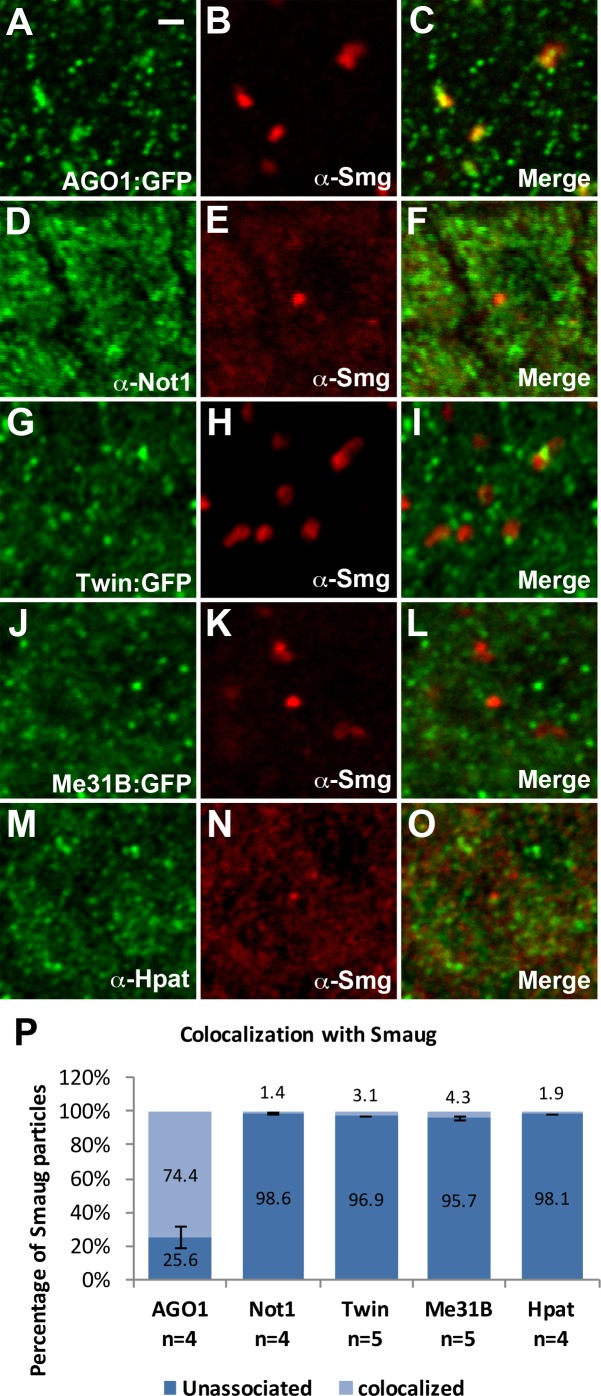
Smg foci colocalize with AGO1. (A-C) Smg foci colocalize with AGO1. (D-I) Smg foci do not colocalize with deadenylation complex components Not1 and Twin. (J-O) Smg does not colocalize with decapping activators Me31B and Hpat. (P) Quantifications of colocalization with Smg foci. A total of 454, 642, 510, 467, and 642 Smg particles were counted respectively for AGO1, Not1, Twin, Me31B, and Hpat double labelings with Smg. The Pearson's correlation coefficient (PCC) value (mean ± SD) for AGO1, Not1, Twin, Me31B, and Hpat double labelings with Smg are .383 ± .160, .039 ± .034, -.005 ± .007, -.005± .005, and .032 ± .022 respectively. All images are from apical sections of cellularizing (stage 5) embryos. Scale bar = 1 μm.

As AGO1 and deadenylation complex proteins do not localize to either GW-bodies or P-bodies, we next investigated whether these proteins associate with RNA binding proteins known to direct early embryonic development. The RNA binding protein Smaug (Smg), which represses and destabilizes maternal RNAs during early embryonic development [33,43,44], has been shown to bind AGO1, Not1, Twin/CCR4, and Me31B [45,46]. Smg is present in large, cytoplasmic foci, typically in close proximity to nuclei [47]. Presumably, these Smg foci represent sites of translationally repressed maternal RNA. We find that Smg foci present during cellularization colocalize with the miRISC complex protein AGO1, as revealed by immunofluorescent staining with an AGO1:GFP protein trap line (Fig 6A–6C and 6P). Gw has been shown to physically interact with AGO1 in *Drosophila* cell culture [11]; however, we do not observe colocalization between AGO1 and Gw, suggesting that Smg structures also do not contain Gw proteins and that these particles are distinct. Smg particles also do not colocalize with deadenylation complex proteins Not1 and Twin (Fig 6D–6I and 6P). Previous analysis of Smg foci indicated that Smg only partially colocalizes with DCP1 during early embryonic development [47]. Likewise we find that Me31B and HPat also do not colocalize with Smg foci, indicating that Smg foci are unlike P-bodies (Fig 6J–6O and 6P). Thus, while Smg biochemically interacts with many decapping and deadenylation proteins, none of these appear to localize to Smg foci.

**Fig 7:**
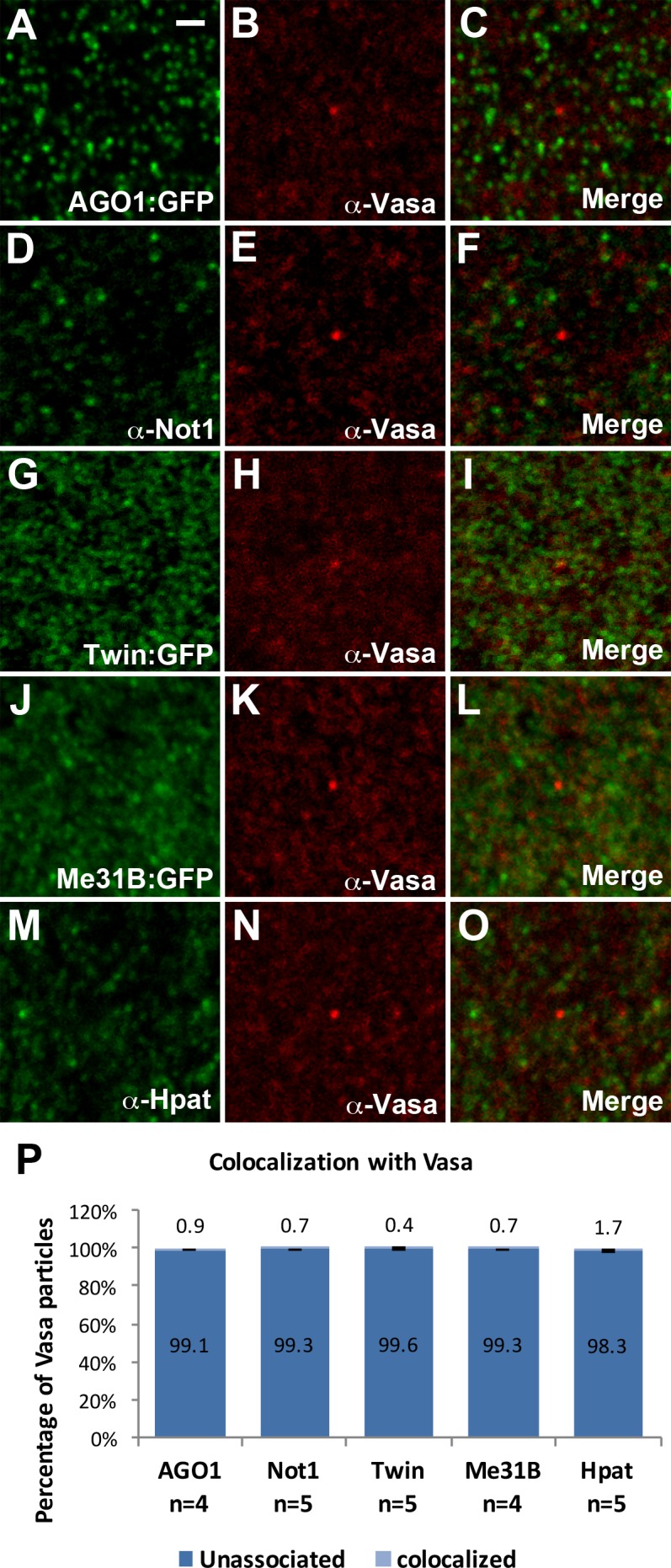
AGO1, deadenylation complex components, and decapping factors are absent from Vasa granules. (A-O) Vasa granules do not colocalize with AGO1 (A-C), deadenylation complex components Not1 and Twin (D-I), or decapping activators Me31B and Hpat (J-O). (P) Quantifications of colocalization with Vasa foci. A total of 542, 548, 730, 548, and 476 Vasa particles were counted respectively for AGO1, Not1, Twin, Me31B, and Hpat double labelings with Vasa. The Pearson's correlation coefficient (PCC) value (mean ± SD) for AGO1, Not1, Twin, Me31B, and Hpat double labelings with Vasa are .001 ± .002, .016 ± .014, -.013 ± .009, .016± .011, and .008 ± .004 respectively. All images are from apical sections of cellularizing (stage 5) embryos. Scale bar = 1 μm.

Early fly embryos also possess RNA-bound particles associated with the Vasa protein. While the Vasa mRNA transcript is uniformly present throughout the embryo, Vasa protein is predominantly expressed in the posterior of the embryo and is highly enriched in the pole plasm [48]. We examined Vasa granules located outside of the pole plasm and observed that RISC, deadenylase, and decapping complex components do not colocalize with Vasa protein (Fig 7A–7P).

In conclusion, our colocalization analysis suggests that the early fly embryo possesses several distinct RNA foci. In addition to P-bodies and GW-bodies, early embryos also contain separate foci that contain Smg/AGO1, Twin, or Not1. All of these granules are also distinct from RNA transport granules that contain the Vasa protein.

### GW-bodies constitute a separate pool of non-translating mRNAs

**Fig 8:**
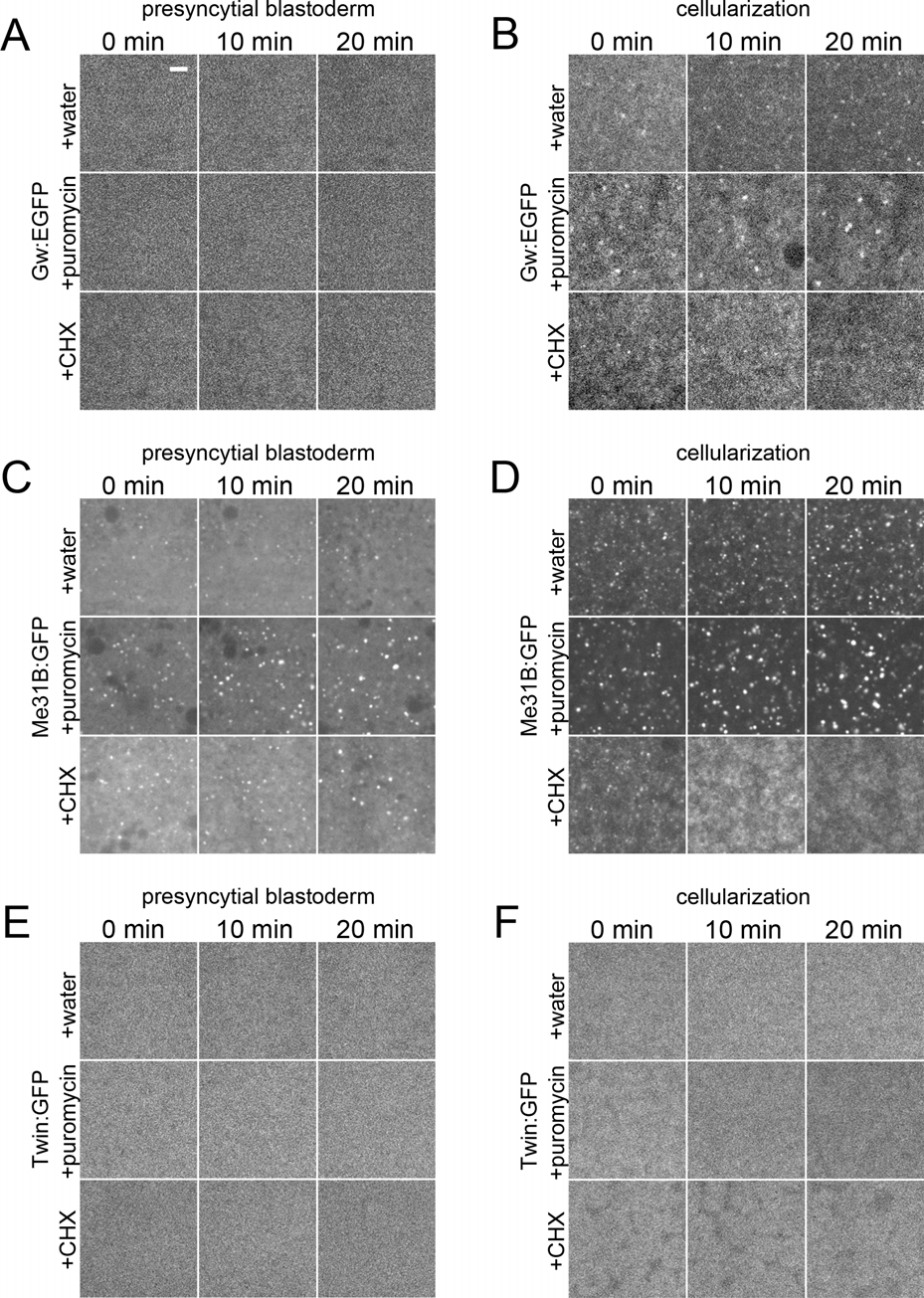
GW-bodies are composed of non-translating RNAs. Live imaging of Gw:EGFP (A,B), Me31B:GFP (C,D), and Twin:GFP (E,F) injected with water, puromycin, or cycloheximide (CHX). (A) Presyncytial blastoderm embryo expressing Gw:EGFP. Puromycin injection does not induce GW-bodies. (B) Cellularizing embryo expressing Gw:EGFP. Puromycin injection induces GW-body formation while cycloheximide injection causes GW-body disintegration. (C) Presyncytial blastoderm embryo expressing ME31B:GFP. Puromycin injection increases P-body size. P-bodies are stable in the presence of cycloheximide. (D) Cellularizing embryo expressing Me31B:GFP. Puromycin injection induces P-body formation while cycloheximide injection causes P-body disintegration. (E) Presyncytial blastoderm embryo expressing Twin:GFP. (F) Cellularizing embryo expressing Twin:GFP. Puromycin injection does not induce the incorporation of Twin into P-bodies (E,F). Scale bar = 5 μm.

P-body size is dependent on the size of the pool of non-translating RNAs [2]. Puromycin, which inhibits protein translation through the premature release of translating RNAs from the ribosome, increases the pool of non-translating RNAs and leads to an enlargement of P-bodies [8,25]. To test if Gw proteins respond to puromycin, we injected *Drosophila* embryos expressing Gw:EGFP either at the presyncytial blastoderm stage (when no GW-bodies are present) or at cellularization and observed GW-body dynamics over a twenty-minute time course. Injection of puromycin into presyncytial blastoderm embryos failed to induce GW-bodies (Fig 8A). Thus, increasing the pool of non-translating RNAs is not sufficient to induce GW-bodies at this stage. However, injection of puromycin into cellularizing embryos causes GW-body enlargement (Fig 8B). These data indicate that GW-bodies are composed of non-translating mRNAs. The failure of puromycin to induce GW-bodies in presyncytial blastoderm embryos suggests that the translation repression mechanism required to regulate sequestration of RNAs into GW-bodies may not be active at this stage. Potentially, this would mean that the microRNA-mediated RNA silencing pathway is not active prior to cellularization. We next tested if P-bodies labeled with Me31B:GFP respond to puromycin injection. We find that puromycin injection increases P-body size during both presyncytial blastoderm and cellularization stages of development (Fig 8C and 8D). Hence, the size of P-bodies and GW-bodies are both dependent on non-translating mRNAs. Finally, we injected puromycin into embryos expressing Twin:GFP. As Twin/CCR4 is a known component of P-bodies in mammalian cells, injection of puromycin should induce the incorporation of Twin into punctate structures. However, we find that puromycin is unable to drive Twin:GFP into P-bodies at either developmental stage (Fig 8E and 8F).

Cycloheximide inhibits translation elongation and consequently traps RNAs in polysomes. This sequestration of RNAs leads to a depletion of non-translating RNAs and consequently to the rapid loss of P-bodies [2,3,24,25]. We injected Me31B:GFP expressing presyncytial blastoderm embryos with cycloheximide and found that P-bodies present at this time point are resistant to cycloheximide (Fig 8C). Thus P-bodies present during early embryogenesis are similar to cycloheximide resistant P-bodies that form in the oocyte during oogenesis [27]. This would be consistent with P-bodies at this stage serving as sites of RNA storage. P-bodies that form during the MBT, however, are not resistant to cycloheximide (Fig 8D). Me31B, which is initially found in discrete cytoplasmic foci during cellularization, redistributes within ten minutes throughout the cytoplasm (Fig 8D). Finally, we injected cycloheximide into Gw:EGFP expressing embryos and found that GW-bodies present at cellularization also disintegrate (Fig 8B). Thus GW-bodies, like P-bodies, are composed of non-translating RNAs.

### New GW-bodies and P-bodies that form at the MBT are derived from maternal RNA

**Fig 9:**
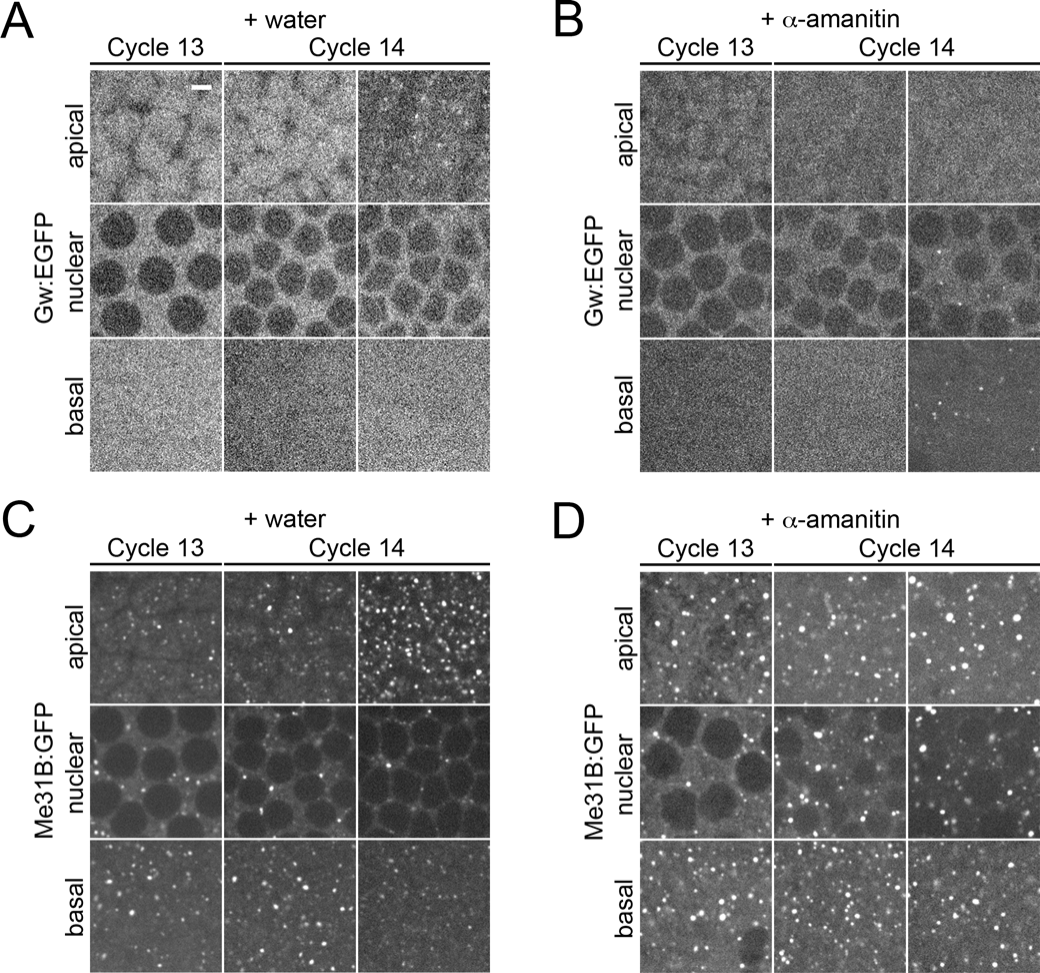
Inhibition of zygotic transcription causes basal mislocalization of GW-bodies, but does not inhibit GW- or P-body formation. Live imaging of water or α-amanitin injected Gw:EGFP and Me31B:GFP embryos. Apical, nuclear, and basal images of embryos during cycles 13 and 14. Cycle 14 images were taken just after completion of cycle 13 (left image) or forty minutes after the completion of cycle 13 (right image). (A) Gw:EGFP expressing embryos injected with water. GW-bodies form apically. (B) Gw:EGFP expressing embryos injected with α-amanitin. GW-bodies form basally. (C) Me31B:GFP expressing embryos injected with water. (D) Me31B:GFP expressing embryos injected with α-amanitin. P-bodies are capable of forming apically to nuclei but appear enlarged. Scale bar = 5 μm.

The activation of zygotic transcription is one of the major hallmarks of the MBT. To determine if new transcription is required for the formation of P-bodies and GW-bodies at the MBT, we injected embryos with the RNA polymerase II inhibitor α-amanitin. We find that disruption of zygotic transcription is unable to block the formation of GW-bodies (Fig 9B). α-amanitin treatment causes GW-bodies to form basally to the nucleus, indicating that zygotic transcription is required to establish proper GW-body distribution (Fig 9B). New P-bodies also form normally when zygotic transcription is blocked (Fig 9D). While capable of forming apically to nuclei, P-bodies that form in the absence of active zygotic transcription appear larger in size (Fig 9D). Altogether, these data indicate that newly formed P-bodies and GW-bodies at the MBT are derived from maternal RNA.

## Discussion

### GW-bodies and P-bodies have different dynamics and distributions in the early *Drosophila* embryo

Our *in vivo* live imaging analysis of GW-bodies and P-bodies indicates that these structures have different spatial and temporal dynamics. While cytoplasmic GW-bodies are mostly absent during early cortical syncytial cycles, P-bodies are present throughout early embryonic development. This is consistent with previous reports that described P-body structures during oogenesis and early embryonic development [27,28]. Here we show that GW-bodies are also present, but localize initially in the nucleus. These nuclear GW-bodies are detectable using live imaging as well as by immunohistochemistry. Consistent with this nuclear localization is the presence of a nuclear localization signal in the Gw sequence [13]. Interestingly, nuclear GW-bodies are depleted as cytoplasmic GW-bodies increase in number, indicating that these two populations of GW-bodies may exist in equilibrium. Similar observations have been made for the P-body component LSm-4, which localizes to Cajal bodies under conditions that limit P-body nucleation [49]. As Gw has been shown to colocalize with LSm-4 in S2 cells [12], one possibility is that nuclear GW-bodies may also overlap with Cajal bodies. The LSm2-8 complex, which functions in RNA splicing within the nucleus, has been shown to be a component of Cajal bodies [50]. Thus, Gw proteins may potentially interact with shared components between the cytoplasmic LSm1-7 complex and the nuclear LSm2-8 complex. Interestingly, Cajal bodies also disassemble and reassemble with the cell cycle [51]. We observed that nuclear GW-bodies form and disintegrate with the cell cycle as well, similar to previous observations of cytoplasmic GW-bodies in mammalian cells [23]. P-bodies, however, are extremely stable during the cortical syncytial cycles, thus further highlighting the differences in dynamics between both structures.

### GW-bodies are distinct from P-bodies

Previous analyses conducted in cell culture indicate that GW-bodies share many of the proteins associated with P-bodies, including DCP1, XRN1 and LSm-4 [10,11,12]. In this report, we conducted an *in vivo* analysis of GW-body constituents using immunohistochemical labelings of endogenous proteins and found that GW-bodies are not associated with typical P-body markers such as DCP1, Me31B, Hpat, Twin, and Not1. Unfortunately, we were unable to determine the presence or absence of LSm proteins to GW-bodies due to the lack of an available antibody. Furthermore, the miRISC component AGO1 was also not observed in Gw foci *in vivo*. Thus, we find that GW-body structures observed in the early embryo differ markedly from those observed in cell culture. These discrepancies may be due to differences in experimental approach. Localization experiments conducted in *Drosophila* S2 cells, which have previously established the existence of a common structure, relied on co-overexpression of Gw with either DCP1, XRN1, LSm-4, or AGO1, [11,12]. Thus, these observations potentially may not reflect endogenous localizations. In fact, co-expression of Gw and AGO1 both *in vivo* and *in vitro* has been shown to induce the recruitment of AGO1 to GW-bodies [11,18]. Potentially these co-overexpression studies reveal transient associations between these proteins and GW-bodies. Our analyses of endogenous proteins indicate that GW-bodies are unique in their composition with regard to P-body or RISC components.

The Gw protein, however, has been shown to physically bind to decapping and deadenylation proteins [11,14,15,16,52]. More importantly, functional assays have implicated decapping and deadenylation downstream of Gw. RNA tethering assays that physically link Gw to RNA reporter constructs demonstrate that Gw promotes RNA decay and translational silencing in both *Drosophila* S2 cells and in a heterologous yeast model [11,53]. Notably, these assays reveal that inhibition of decapping or deadenylation using RNA knockdown suppresses Gw-dependent RNA decay without affecting translational silencing [11,16]. We find that neither deadenylation regulators nor decapping factors localize to Gw-bodies *in vivo*, suggesting that GW-bodies are not sites of RNA decay. Thus, the RNA metabolic functions associated with Gw may occur outside of GW-bodies themselves. Recently, decapping, deadenylation, and AGO1/Gw proteins have been localized to actively translating ribosomes [54]. Therefore, polysomes may potentially serve as alternative sites for RNA decay. Potentially, GW-bodies may exist only as sites of sequestered non-translating RNAs. We also find that Smg foci do not colocalize with components of the CCR4-Not deadenylation complex or with decapping factors, despite the biochemical associations between Smg and these proteins. Thus translationally repressed, sequestered RNAs may not associate in general with decapping or deadenylation effectors.

Despite compositional differences, both Gw-bodies and P-bodies are similar in that they exist in equilibrium with actively translating pools of RNAs. We find that artificially increasing the pool of non-translating RNAs using puromycin injection enhances the size of both P-bodies and GW-bodies. Thus GW-bodies, like P-bodies, represent a variety of non-translating RNAs. However, the release of RNAs from polysomes is not sufficient to induce GW-body formation during the presyncytial blastoderm stage of development. Several explanations can account for this failure to incorporate Gw into Gw-bodies. One possibility is that the Gw protein at this stage may not be associated with RNA. Another possibility is that a critical factor required for the incorporation of Gw into GW-bodies is missing from embryos at this stage or that the presyncytial embryo might express a repressor that blocks GW-body formation. Potentially, the translation inhibition mechanism that induces GW-body formation or an appropriate RNA substrate may be absent.

### *Drosophila* P-bodies are enriched for decapping factors

P-bodies have been proposed to act as sites of RNA decay and are associated with various forms of RNA turnover including 5’-3’ exonucleolytic decay and nonsense mediated decay [2,3,6,55]. In yeast, P-bodies are associated with the 5’-3’ exonuclease XRN1 as well as decapping factors DCP1 and DCP2 and decapping regulators Pat1/Hpat, Dhh1/Me31B, and the LSm1-7 complex [2]. In addition to these regulators of decapping and degradation, proteins that are functionally implicated in deadenylation also localize to P-body structures. CCR4, which serves as the catalytic component of the CCR4-NOT deadenylation complex, is weakly present in yeast P-bodies [2]. The association between CCR4 and P-bodies has been shown to increase upon stress signaling induced by glucose withdrawal [56]. On the other hand, the localization of Not1 to P-bodies has only been shown under conditions of impaired decapping [57]. Such observations suggest that deadenylation proteins only transiently associate with P-bodies in yeast. P-bodies in higher eukaryotes, however, are more heterogenous in their protein composition. Components that regulate deadenylation such as CCR4/Twin appear to be constitutive components of P-bodies in higher organisms [3,24]. Furthermore, other components such as regulators of the miRNA pathway, including the argonaute proteins and Gw/GW182, have been shown to localize to P-body-like structures [10,17,21]. Our analysis of P-bodies in *Drosophila* embryos suggests that these structures are enriched primarily for decapping factors. Unlike P-bodies observed in mammalian cells, neither deadenylation complex factors nor miRNA regulators localize to DCP1 foci. These data are consistent for a role for P-bodies in RNA destabilization via decapping. Indeed, P-bodies that form at the midblastula transition appear to be important for the destabilization of maternal RNAs such as *bicoid*, *oskar*, and *twine* [27,58].

Interestingly, we were not able to induce the recruitment of Twin to P-bodies using puromycin treatment. These results indicate that deadenylation potentially occurs outside of these structures. Another possibility is that components that regulate deadenylation only incorporate into P-bodies upon stress. This is in line with many observations that have implicated environmental stress in the regulation of P-body size and number. In unicellular organisms such as budding yeast, changes in nutrient availability, osmotic pressure, ultraviolet light exposure, or low pH trigger mobilization of translating RNAs into P-bodies [1,59]. Similar roles for P-bodies have also been observed in multicellular organisms. Oogenesis in *Drosophila* is regulated by nutrient availability, and starvation of female flies induces a mid-oogenesis checkpoint that leads to the degradation of late stage egg chambers and an arresting of pre-vitellogenic egg chambers [60,61]. This nutrient withdrawal induces P-body formation in arrested nurse cells, which appears to be critical for maintaining oocyte viability [62]. Thus *in vivo*, P-body formation is also associated with RNA storage during stress-induced cellular quiescence. Movement of RNAs into P-bodies appears to play a protective function in yeast as well as cells carrying deletion mutations in either LSm1, Pat1 or Dhh1, all of which encode core P-body components, lose viability when subcultured following prolonged nutrient deprivation [63,64]. Potentially stress signaling pathways may be required for the recruitment of deadenylation factors into P-bodies.

### New GW-bodies and P-bodies form at the midblastula transition

The importance of Gw at the MBT has previously been documented as *gw* mutant embryos arrest at this stage and fail to undergo cellularization [12]. Interestingly, both GW-bodies and P-bodies form in large numbers during cellularization. However, inhibition of transcription at the MBT using α-amanitin indicates that zygotic gene activation is not required for the formation of new P-bodies and GW-bodies. Therefore, these new GW-bodies and P-bodies are most likely derived from maternal RNAs. This is consistent with previous reports that indicate that P-bodies play an important role in maternal RNA turnover at the MBT [58]. However, the mechanism that governs the formation of GW-bodies at this stage remains a mystery. In *Drosophila*, miR-309 cluster is zygotically expressed at the MBT to promote maternal RNA turnover [65]. As Gw plays a major functional role in miRNA-mediated gene silencing, we expected that α-amanitin treatment would inhibit miRNA dependent degradation of maternal RNA and that we would observe a concomitant effect of GW-body formation. Knockout of zygotic transcription, however, causes no effect on GW-body formation. Thus, nucleation of GW-bodies during the MBT is potentially not dependent on zygotic miRNA expression. The enlargement of P-bodies after the inhibition of zygotic gene activation suggests that efficient turnover of maternal RNAs may be compromised. This may reflect a loss of miR-309 cluster expression. GW-bodies, on the other hand, may form to shield a subset maternal RNAs from MBT-associated degradation.

## Conclusion

Altogether, these data indicate that GW-bodies and P-bodies represent two separate pools of non-translating RNAs. GW-bodies have a different spatial-temporal regulation, biochemical composition, and subcellular distribution mechanism to P-bodies. Future characterizations of the RNA contents of GW-bodies will provide valuable insights into the functional role of these structures. Furthermore, identification of RNAs present in GW-bodies may disclose RNAs important for regulating developmental processes such as cellularization.

## Supporting Information

S1 Fig EGFP tagged GW-bodies in the early *Drosophila* embryo. (A-F) GW-bodies in cellularizing (stage 5) embryos labeled with anti-GFP and anti-Gw. (A-C) GW-bodies are present apical to nuclei. The Pearson's correlation coefficient (PCC) value (mean ± SD) for Gw:EGFP and Gw is .571 ± .057, n = 6. (D-F) Sagittal view of cellularizing embryos double labeled to detect Gw:EGFP and the endogenous Gw protein. (G-I) Nuclear GW-bodies are present in all syncytial cycle 10 (G), cycle 11 (H), and cycle 12 (I) nuclei.
(TIF)

S2 Fig Deadenylation complex proteins at cycle 14. (A-C) Not1 and Twin do not colocalize. (D-F) Basal Not1 foci are associated with actin bundles, marked by phalloidin. Scale bar = 5 μm.
(TIF)
