# Supplementary figures and images for: GW-Bodies and P-Bodies Constitute Two Separate Pools of Sequestered Non-Translating RNAs
Source: PLoS One. 2016 Mar 1;11(3):e0150291. doi: 10.1371/journal.pone.0150291 (PMC4773245; doi:10.1371/journal.pone.0150291)

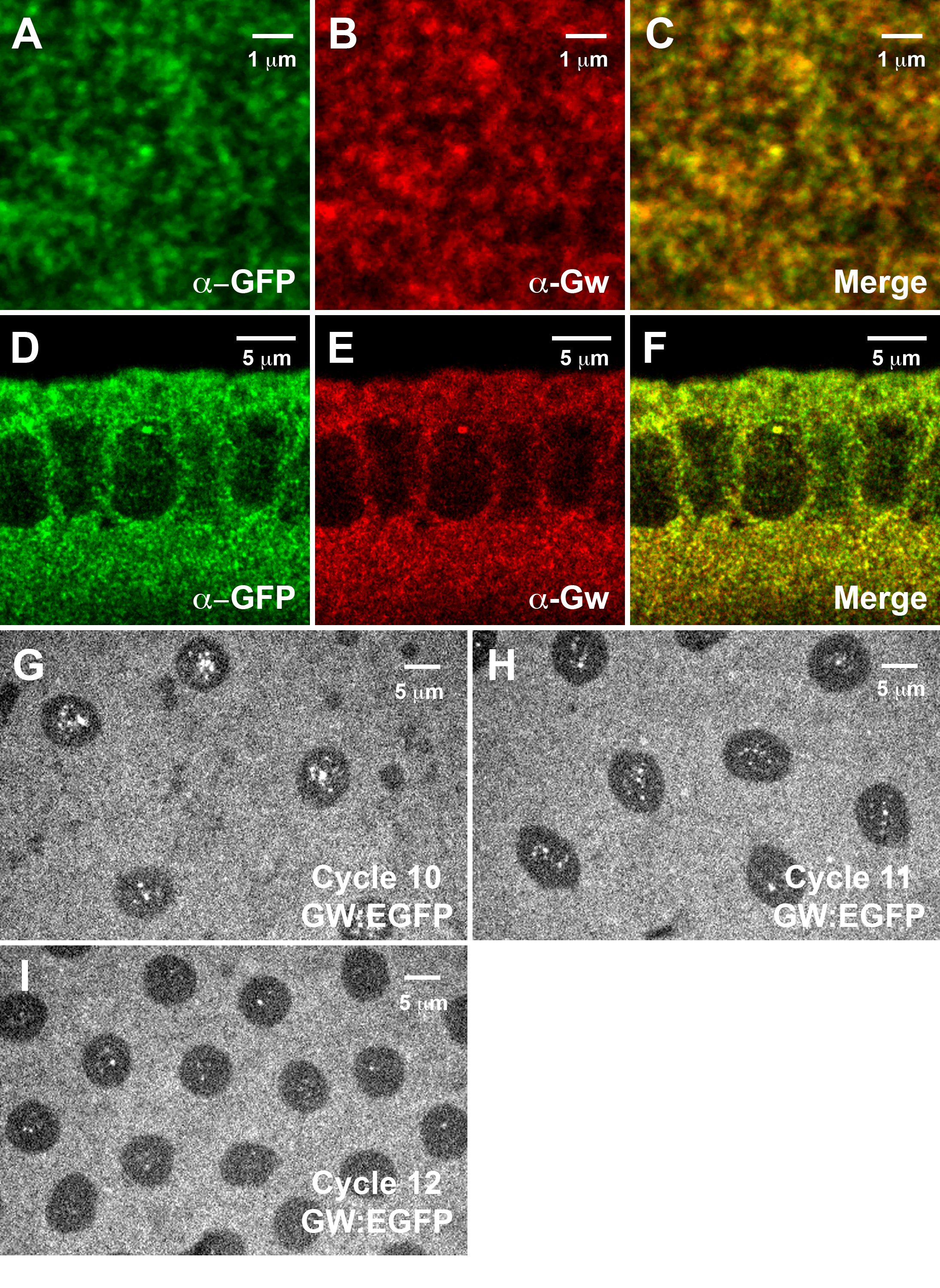

Supplement: S1 Fig — (A-F) GW-bodies in cellularizing (stage 5) embryos labeled with anti-GFP and anti-Gw. (A-C) GW-bodies are present apical to nuclei. The Pearson's correlation coefficient (PCC) value (mean ± SD) for Gw:EGFP and Gw is .571 ± .057, n = 6. (D-F) Sagittal view of cellularizing embryos double labeled to detect Gw:EGFP and the endogenous Gw protein. (G-I) Nuclear GW-bodies are present in all syncytial cycle 10 (G), cycle 11 (H), and cycle 12 (I) nuclei. (TIF) [file pone.0150291.s001.tif]

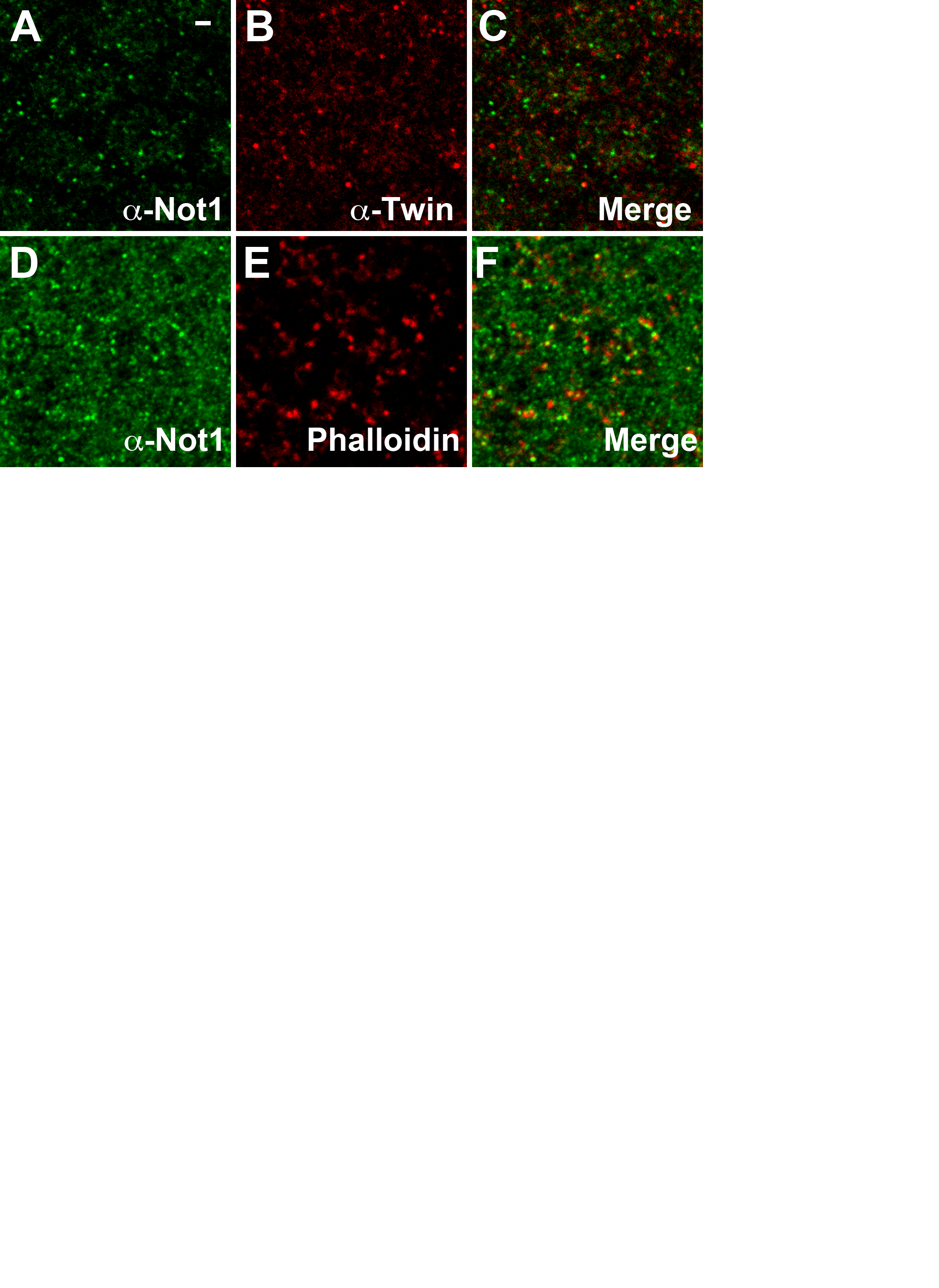

Supplement: S2 Fig — (A-C) Not1 and Twin do not colocalize. (D-F) Basal Not1 foci are associated with actin bundles, marked by phalloidin. Scale bar = 5 μm. (TIF) [file pone.0150291.s002.tif]
